# Supplementary figures and images for: Leishmania survives by exporting miR-146a from infected to resident cells to subjugate inflammation
Source: Life Sci Alliance. 2022 Feb 24;5(6):e202101229. doi: 10.26508/lsa.202101229 (PMC8881743; doi:10.26508/lsa.202101229)

Fig 1B

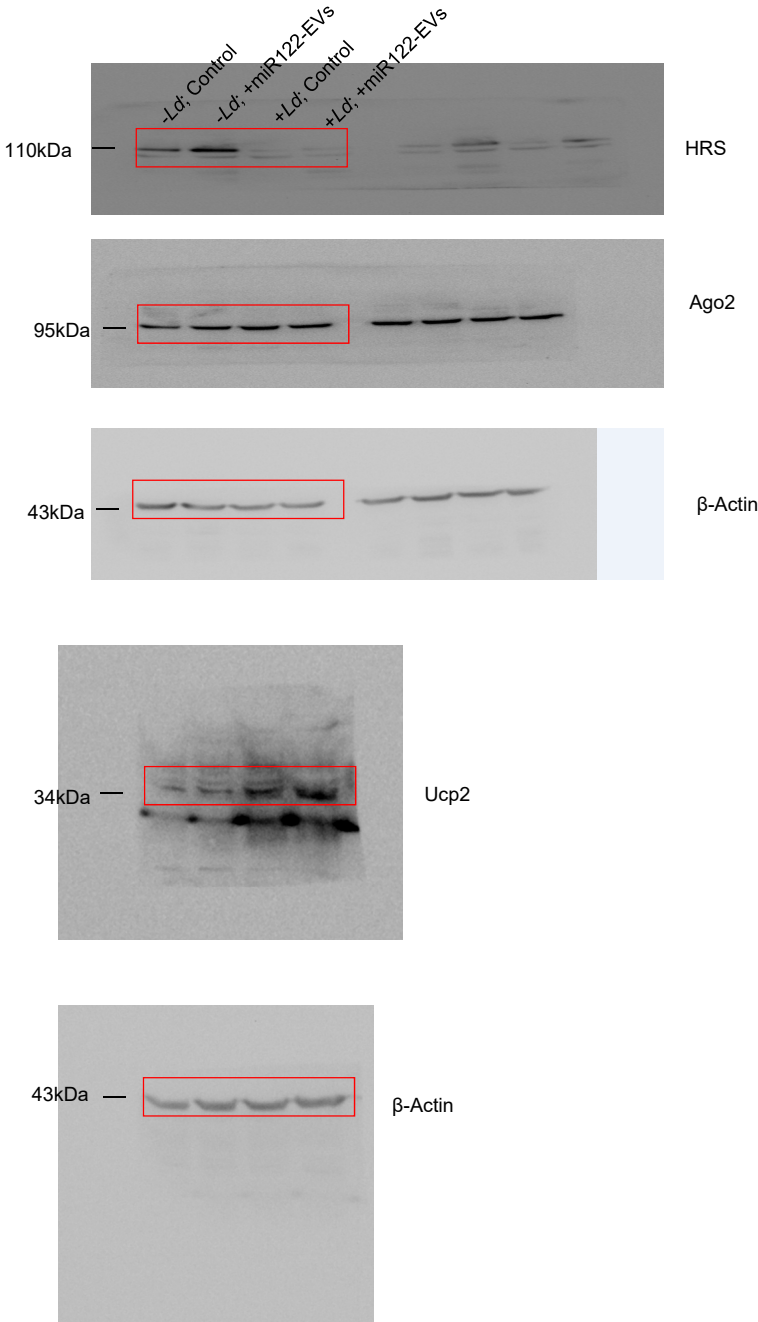

Supplement: Supplementary file 1 [file LSA-2021-01229_SdataF1.pdf]

## SUPPLEMENTARY S2 C

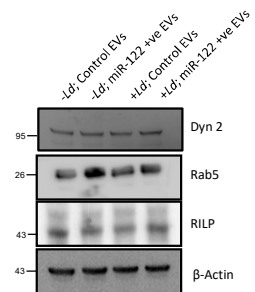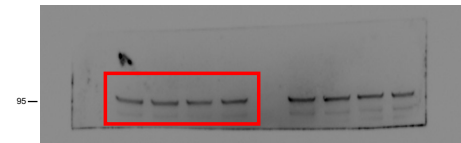

Dynamin2

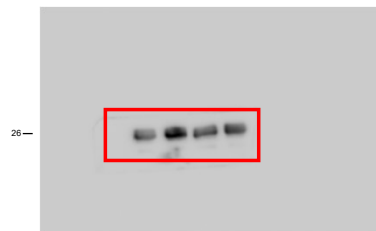

Rab5a

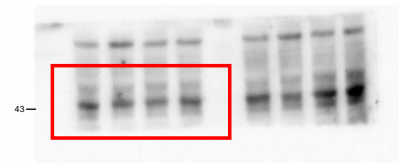

RILP

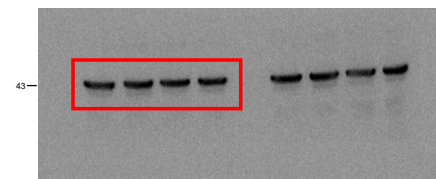

β-Actin

Supplement: Supplementary file 2 [file LSA-2021-01229_SdataFS2.pdf]

Fig 3D

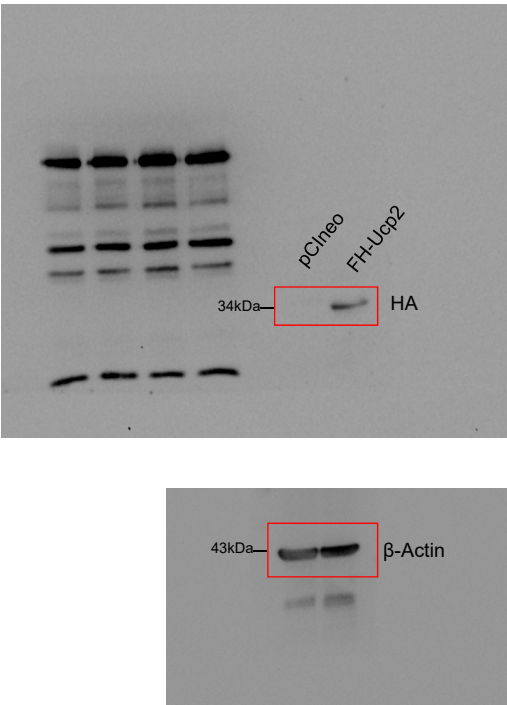

Fig 3F

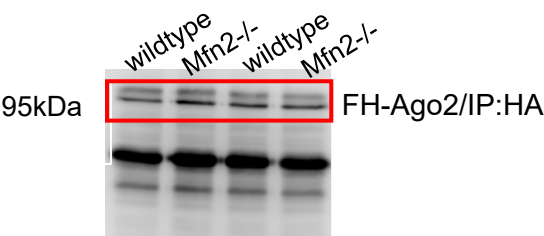

Supplement: Supplementary file 3 [file LSA-2021-01229_SdataF3.pdf]

**Fig 4B**

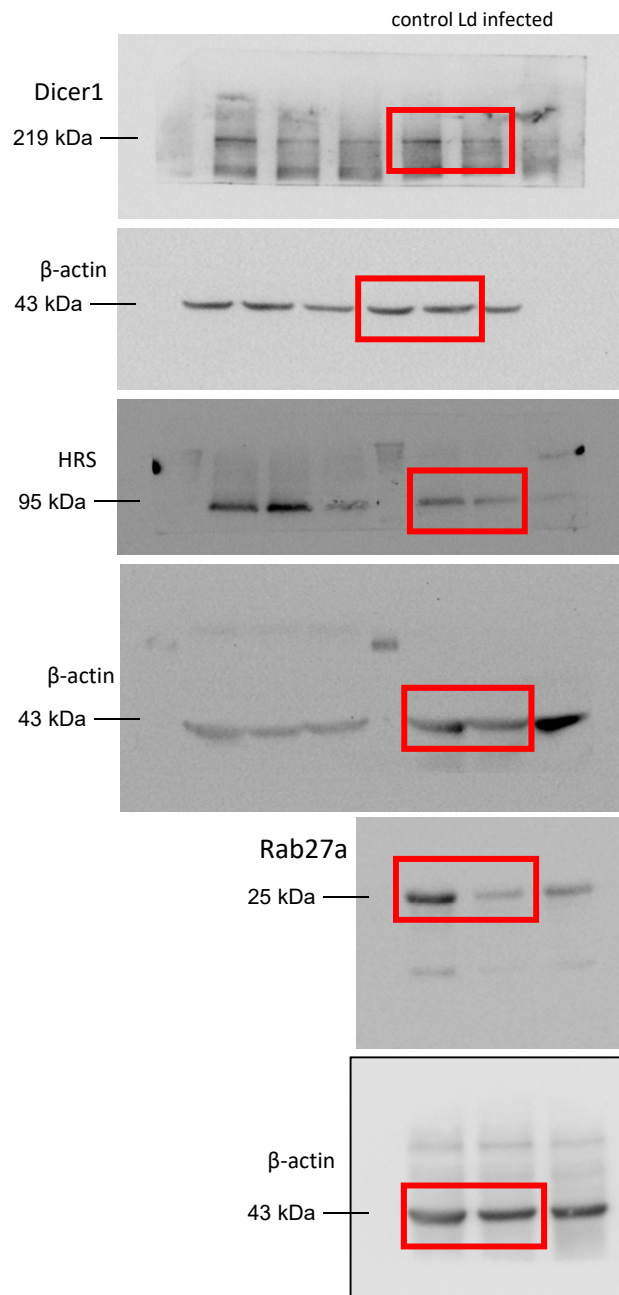

**Fig 4D**

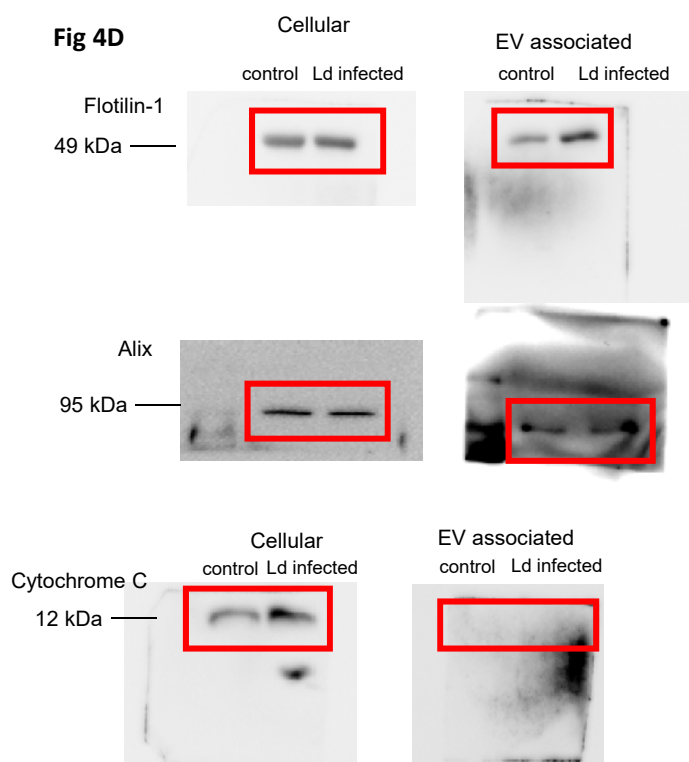

**Fig 4H**

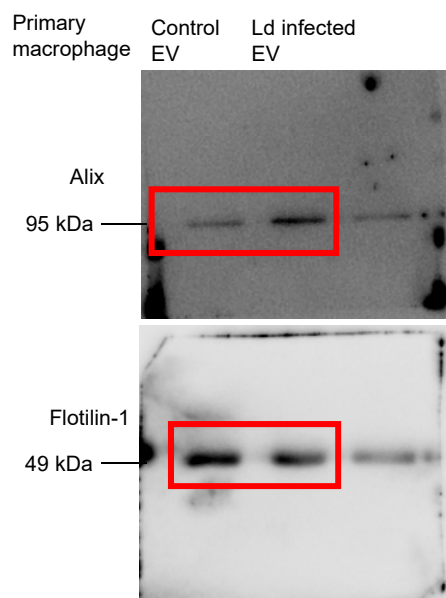

Supplement: Supplementary file 4 [file LSA-2021-01229_SdataF4.pdf]

Fig S5 E

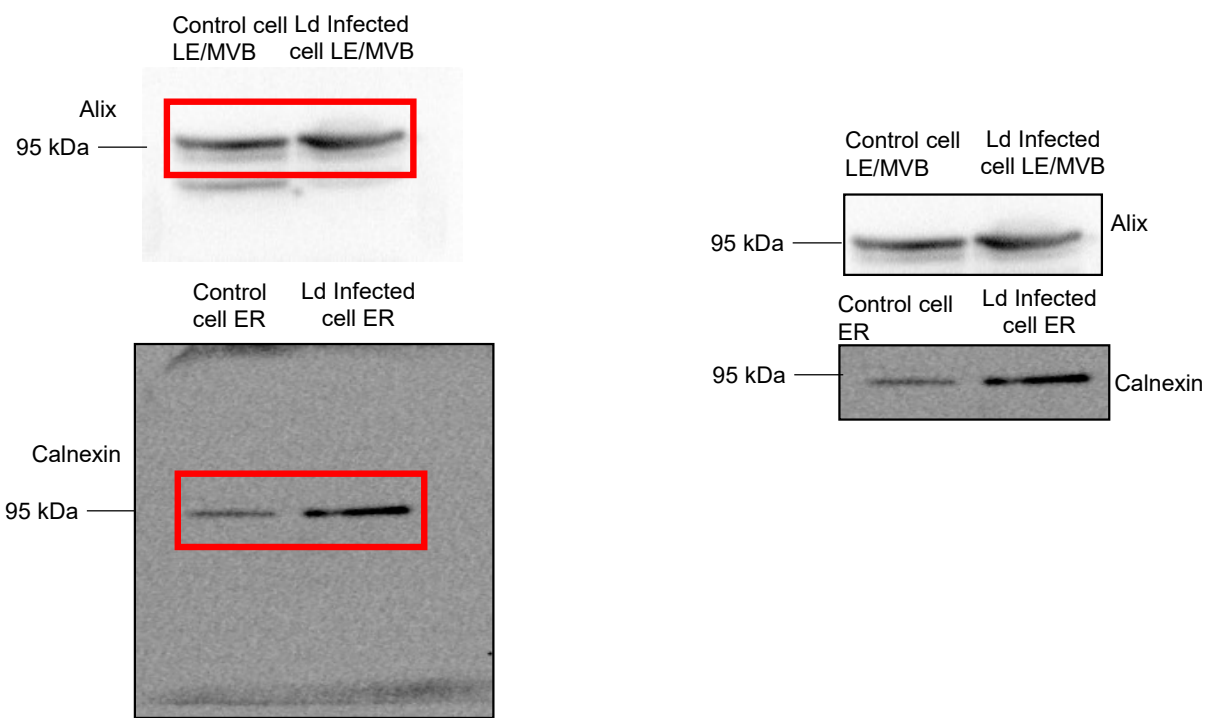

Fig S5 H

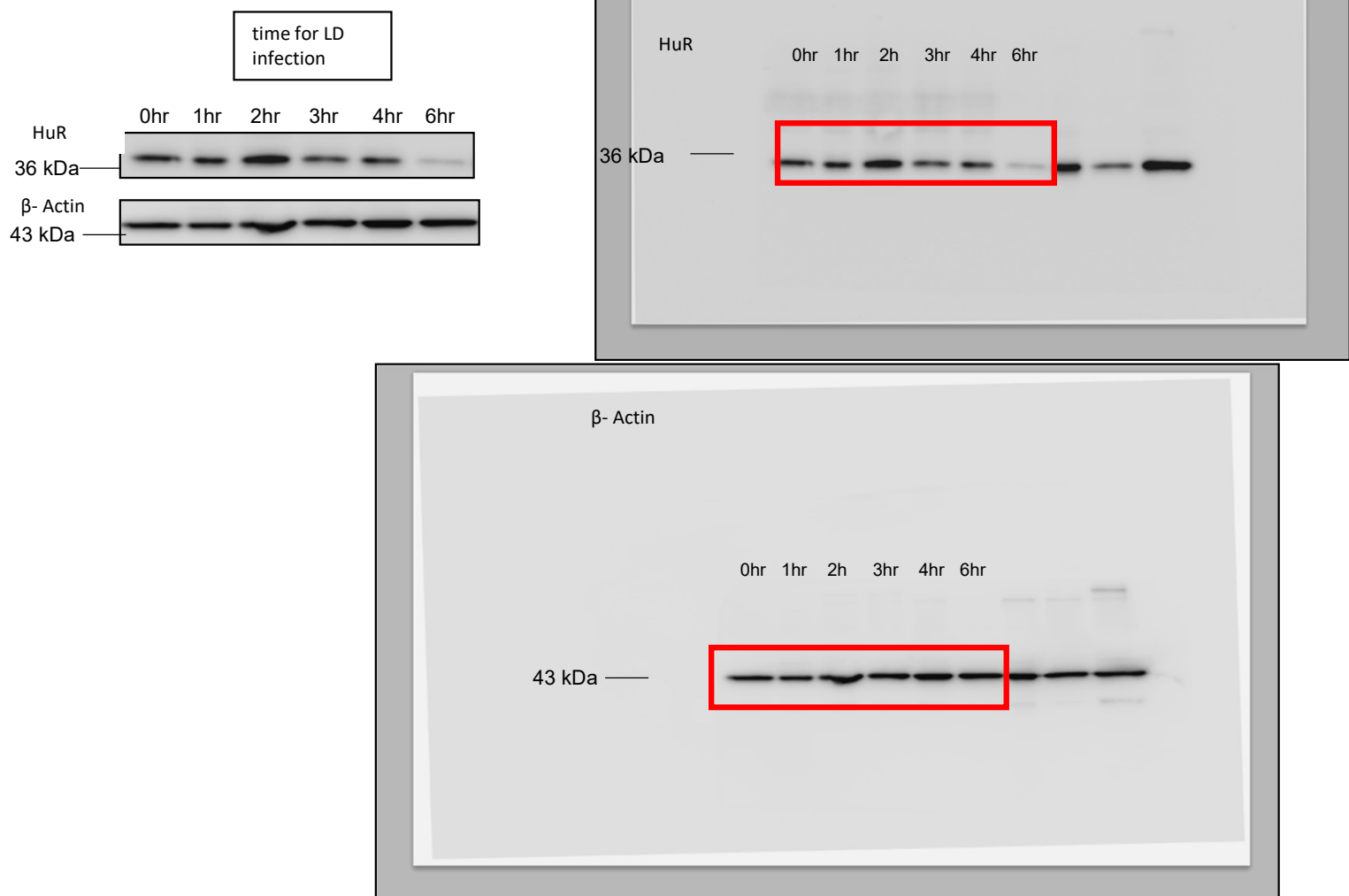

Figure S5 I

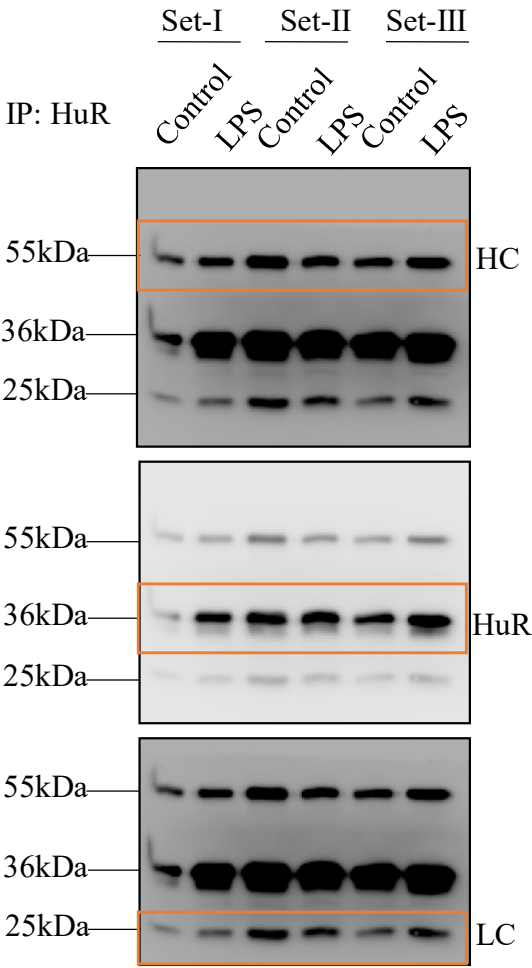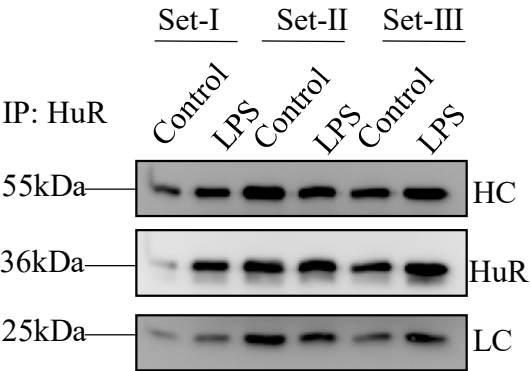

Figure S6 C

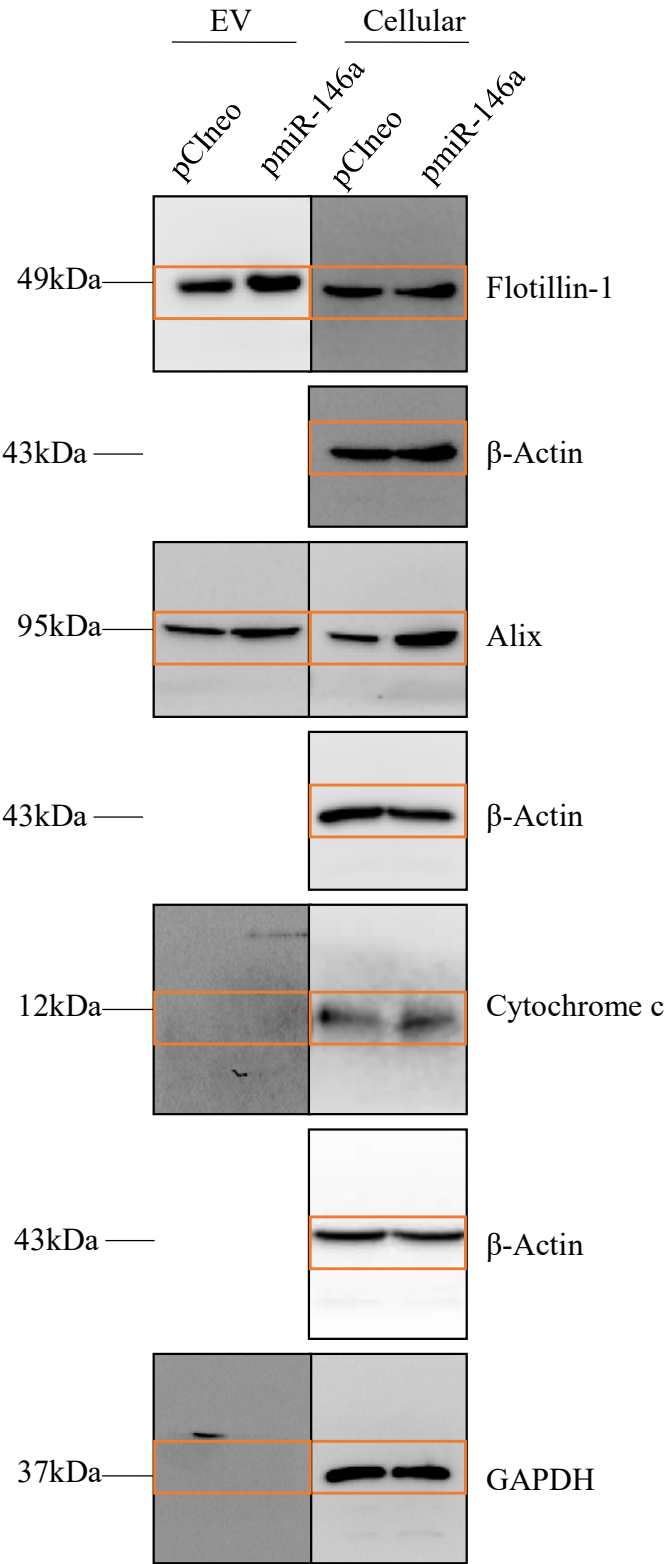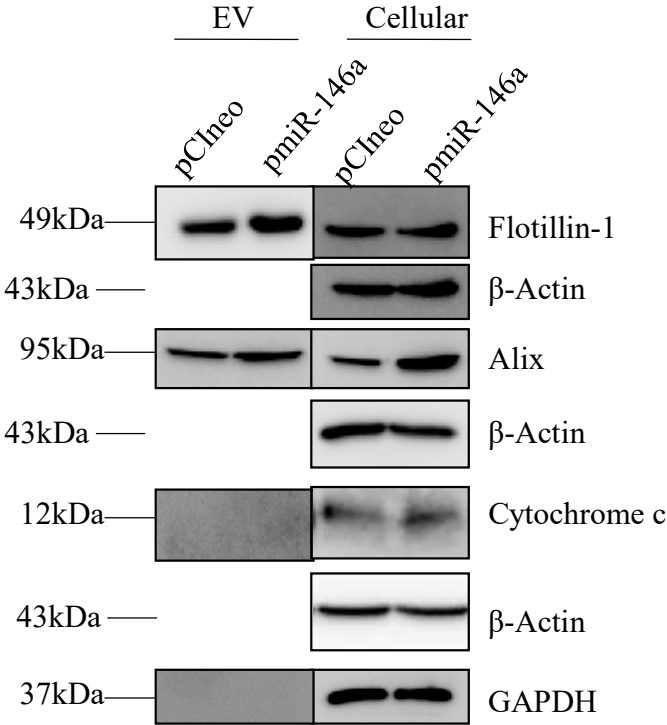

Supplement: Supplementary file 8 [file LSA-2021-01229_SdataFS5.pdf]

**Fig 5F**

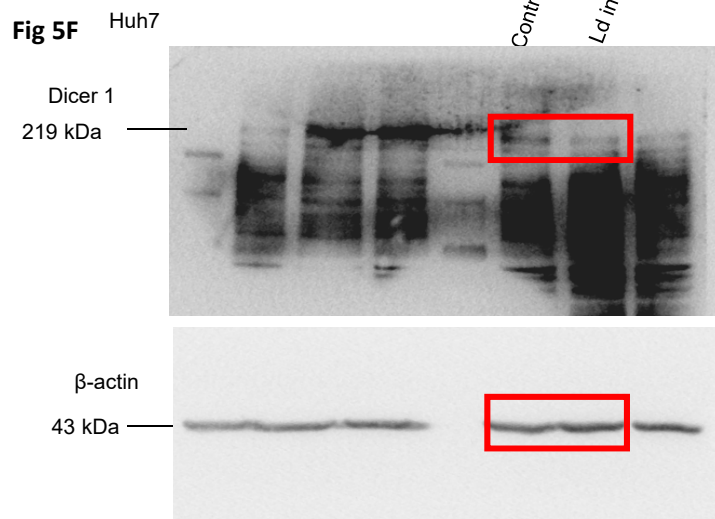

**Fig 5G**

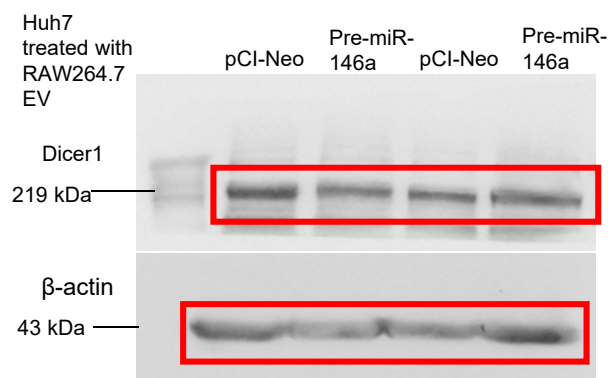

**Fig 5H**

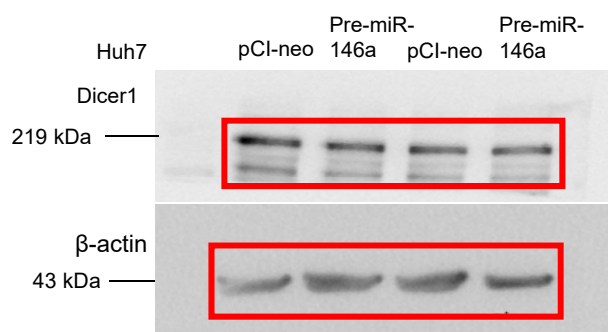

**Fig 5I**

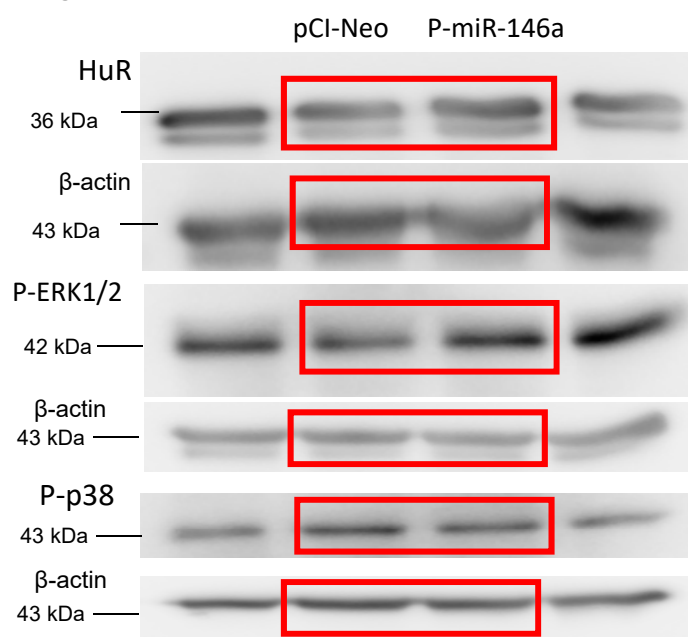

**Fig 5K**

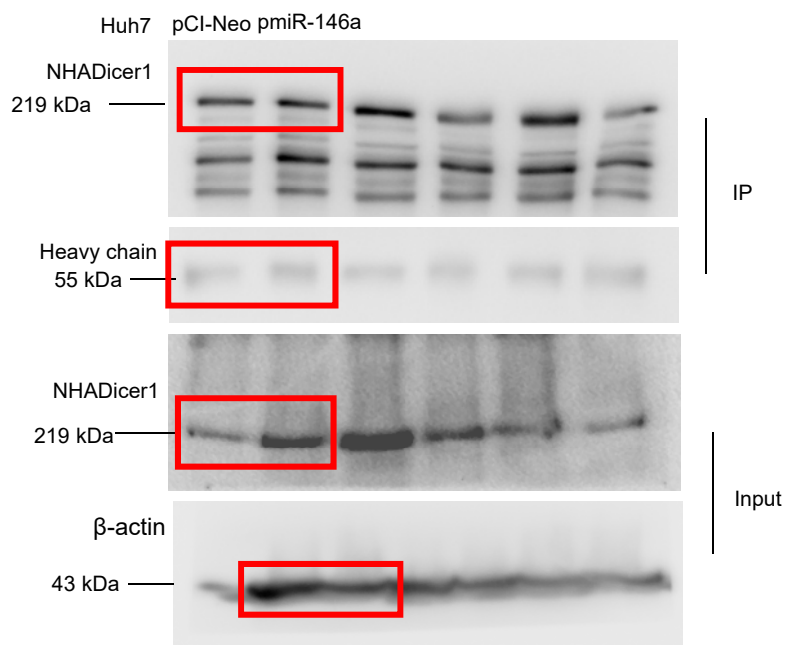

Supplement: Supplementary file 9 [file LSA-2021-01229_SdataF5.pdf]

**Fig 6E**

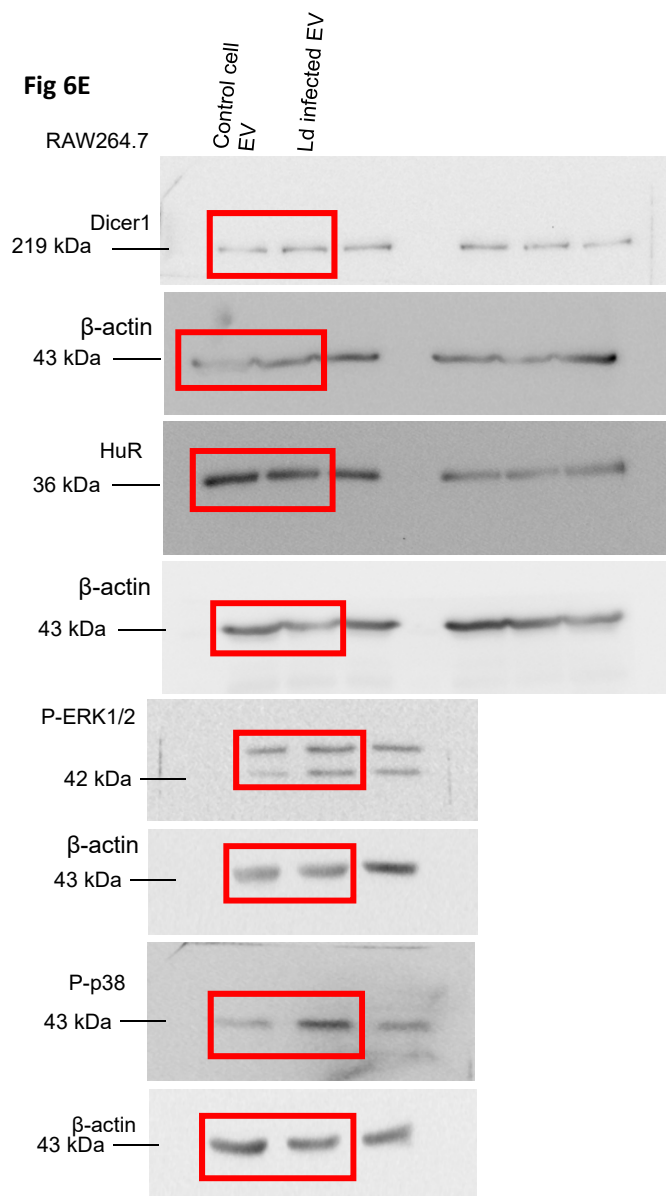

Supplement: Supplementary file 10 [file LSA-2021-01229_SdataF6.pdf]

Fig. 7 A

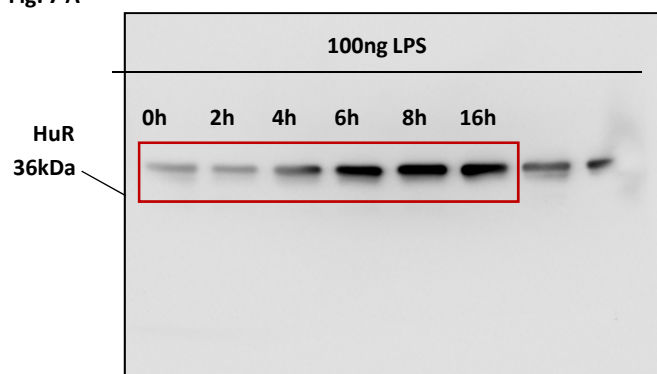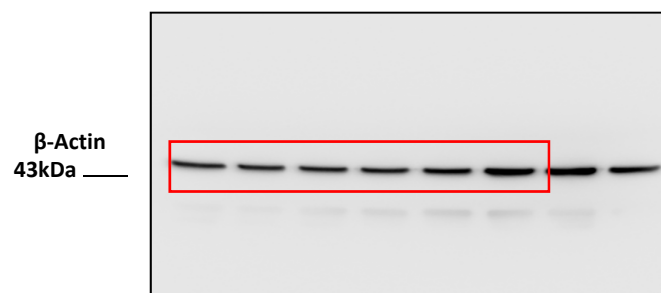

Figure 7 C

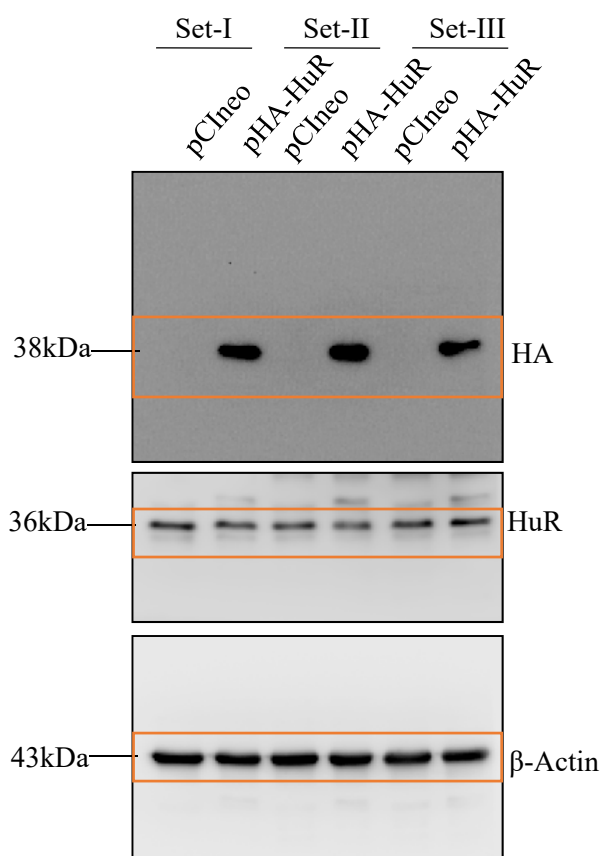

Figure 7 E

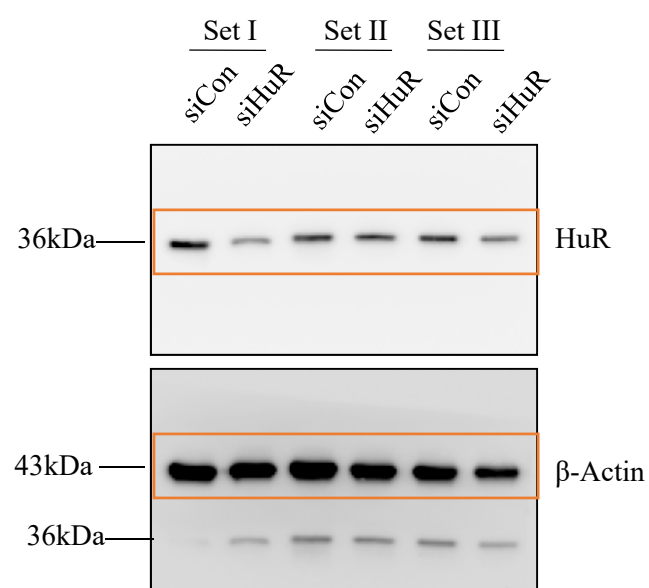

Figure 7G

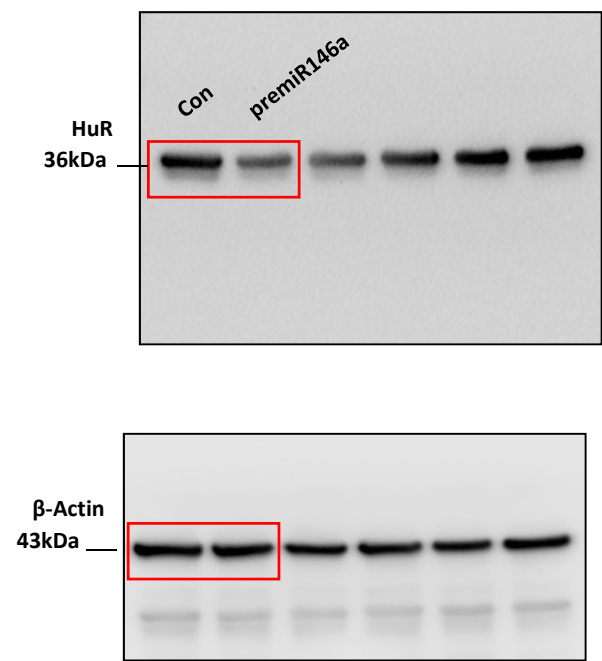

Figure 7 H

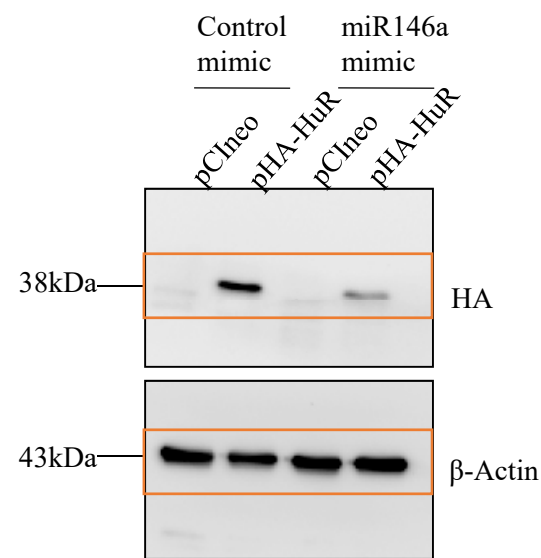

Figure 7 M

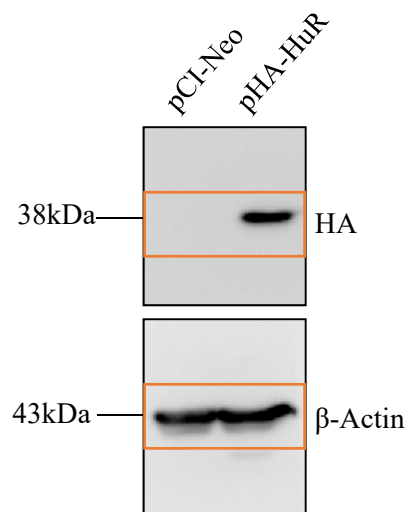

Supplement: Supplementary file 11 [file LSA-2021-01229_SdataF7.pdf]
