# Supplementary material for: Leishmania survives by exporting miR-146a from infected to resident cells to subjugate inflammation
Source: Life Sci Alliance. 2022 Feb 24;5(6):e202101229. doi: 10.26508/lsa.202101229 (PMC8881743; doi:10.26508/lsa.202101229)
Supplement: Supplementary file 6 [file LSA-2021-01229_TableS2.docx]

**Table S2 Comparative analysis of common proteins present in control as well as infected EVs based on their score of relative abundance and function. Relative score for infected EV is calculated and compared to control EV**

| **SL. NO** | **Proteins** | **EV type** | **Function** | **Relative score for infected EV** |
| --- | --- | --- | --- | --- |
| 1 | GRP78 | Control &infected | Molecular Chaperon involve in protein folding | 0.76 |
| 2 | Serum Albumin | Control & infected | Carrier protein | 0.96 |
| 3 | Prothrombin | Control & infected | Coagulation factor | 0.8 |
| 4 | Haemoglobin subunit alpha | Control & infected | oxygen carrier | 0.5 |
| 5 | Inter-alpha-trypsin inhibitor heavy chain H3 | Control & infected | Prorease inhibitor | 0.9 |
| 6 | Gelsolin | Control & infected | Actin binding protein | 1.13 |
| 7 | Complement C3 | Control & infected | Activator of classical & alternative pathways: Elimination of immunocomplexes | 0.39 |
| 8 | Inter-alpha-trypsin inhibitor heavy chain H2 | Control & infected | Protease inhibitor | 0.51 |
| 9 | Alpha-2-HS-glycoprotein | Control & infected | Carrier protein, role in endocytosis. Brain development, bone tissue formation | 0.61 |
| 10 | Actin, cytoplasmic 1 | Control & infected | Cytoskeletal protein | 0.73 |
| 11 | Profilin-1 | Control & infected | Regulate actin polymerization | 1.66 |
| 12 | AN1-type zinc finger protein 1 | Control & infected | Regulates cytoplasmic stress granule turnover | 1.025 |
| 13 | Protein S100-A6 | Control & infected | Calcium binding proteins | 1.69 |
| 14 | Murinoglobulin-1 | Control & infected | Protease inhibitor | 0.88 |
| 15 | NADH dehydrogenase [ubiquinone] 1 alpha subcomplex assembly factor 4 | Control & infected | Involve in Electron Transport Chain | 0.9 |
| 16 | Adenosylhomocysteinase | Control & infected | enzyme | 1.7 |
| 17 | Alpha-2-macroglobulin-P | Control & infected | antiprotease | 1.19 |
| 18 | Elongation factor 1-alpha 1 | Control & infected | Translation | 6.58 |
| 19 | Aurora kinase A | Control & infected | Regulates cell cycle by regulating mitotic spindles | 1.07 |
| 20 | FtsJ methyltransferase domain-containing protein 1 | Control & infected | Methltransferase, RNA methylation, Ribosome niogenesis | 1.16 |
