# Supplementary material for: Leishmania survives by exporting miR-146a from infected to resident cells to subjugate inflammation
Source: Life Sci Alliance. 2022 Feb 24;5(6):e202101229. doi: 10.26508/lsa.202101229 (PMC8881743; doi:10.26508/lsa.202101229)
Supplement: Supplementary file 7 [file LSA-2021-01229_TableS3.docx]

**Table S3 Comparative analysis of proteins present in control and infected EVs based on their score of relative abundance.**

| SL No. | Proteins | EV type | Function | score |
| --- | --- | --- | --- | --- |
| 1 | Heat shock-related 70 kDa protein 2 | Control | Molecular chaperon | 100.82 |
| 2 | Heat shock cognate 71 kDa protein | Infected | Protein folding | 126.14 |
| 3 | V-type proton ATPase 16 kDa proteolipid subunit | Control | Protein sorting, receptor mediated endocytosis, synaptic vesicle proton gradient generation | 61.25 |
| 4 | Histone H3.3 | Control | Genome integrity | 32.24 |
| 5 | Zinc finger homeobox protein 3 | control | Transcriptonal regulator | 27.82 |
| 6 | Keratin, type II cytoskeletal 74 | control | Hair formation | 27.75 |
| 7 | Potassium voltage-gated channel subfamily KQT member 5 | control | Ion transport | 24.93 |
| 8 | Protein unc-80 homolog | control | Cation channel activity | 24.93 |
| 9 | Calreticulin | control | Calcium binding | 24.65 |
| 10 | MAP kinase-activating death domain protein | control | Guanyl nucleotide exchange factor | 23.84 |
| 11 | Proteasome subunit beta type-10 | control | Peptidase activity | 21.83 |
| 12 | Pyruvate kinase isozymes M1/M2 | infected | glycolysis | 282.66 |
| 13 | Alpha-enolase | infected | glycolysis | 198.56 |
| 14 | CD9 antigen | infected | Tetraspanin protein | 179.48 |
| 15 | Guanine nucleotide-binding protein G(I)/G(S)/G(T) subunit beta-2 | infected | G-protein formation | 89.33 |
| 16 | Aminopeptidase N | infected | Peptidase activity, role in antigen presentation, angiogenesis | 43.62 |
| 17 | Sodium/potassium-transporting ATPase subunit alpha-1 | infected | Ion transport channel formation | 43.47 |
| 18 | Moesin | infected | T cell & B cell homeostasis, self tolerence | 39.66 |
| 19 | 4F2 cell-surface antigen heavy chain | infected | Amino acid transporter | 36.50 |
| 20 | Thrombospondin-1 | infected | Adhesive glycoprotein | 35.67 |
| 21 | Collagen alpha-1(VI) chain | infected | Tissue integrity | 34.38 |
| 22 | Talin-1 | infected | Linking integrin with actin | 32.77 |
| 23 | L-lactate dehydrogenase C chain | infected | Pyruvate metabolism | 25.39 |
| 24 | Angiopoietin-related protein 4 | infected | Angiogenesis, lipid metabolism | 24.90 |
| 25 | Pre-mRNA-splicing factor ISY1 homolog | infected | mRNA splicing | 24.65 |
| 26 | Long-chain-fatty-acid--CoA ligase 1 | infected | Lipid metabolism | 23.11 |
| 27 | Amyloid beta A4 precursor protein-binding family A member 3 | infected | Protein transport | 22.14 |
| 28 | DNA-binding protein inhibitor ID-4 | infected | Transcription regulation | 20.94 |
