# Supplementary material for: Leishmania survives by exporting miR-146a from infected to resident cells to subjugate inflammation
Source: Life Sci Alliance. 2022 Feb 24;5(6):e202101229. doi: 10.26508/lsa.202101229 (PMC8881743; doi:10.26508/lsa.202101229)
Supplement: Supplementary file 12 [file LSA-2021-01229_TableS4.docx]

**Table S4 Details of miRNA assays used for Taqman based quantification**

| **miRNA** | **Assay ID** |
| --- | --- |
| mouse miR-155 | 002571 |
| human miR-146a | 000468 |
| human miR-21 | 000397 |
| human miR-16 | 000391 |
| human miR-125b | 000449 |
| human miR-122 | 000445 |
| U6 | 001973 |
