# Supplementary material for: Leishmania survives by exporting miR-146a from infected to resident cells to subjugate inflammation
Source: Life Sci Alliance. 2022 Feb 24;5(6):e202101229. doi: 10.26508/lsa.202101229 (PMC8881743; doi:10.26508/lsa.202101229)
Supplement: Supplementary file 13 [file LSA-2021-01229_TableS5.docx]

**Table S5 Details of mRNA primers used for SYBR-Green based quantification**

| Target | 5’ Forward Primer 3’ | 5’ Reverse Primer 3’ |
| --- | --- | --- |
| TNF-α | GTCTCAGCCTCTTCTCATTCC | TCCACTTGGTGGTTTGCTA |
| IL-1β | GACCTTCCAGGATGAGGACA | CCTTGTACAAAGCTCATGGAG |
| IL-10 | TGCTAACCGACTCCTTAATGC | ATCACTCTTCACCTGCTCCAC |
| Pre-miR-122 | CCTTAGCAGAGCTGTGGAG | GCCTAGCAGTAGCTATTTAG |
| CAT-1 | GCCGCCGGCTTGGATTCTGA | CCCCGAGGGCCACCAGATCA |
| Albumin | TGCTTTTTCCAGGGGTGTGTT | TTACTTCCTGCACTAATTTGGC |
| Clec-4f | GAGGCCGAGCTGAACAGAG | TGTGAAGCCACCACAAAAAGAG |
| MyD88 | TCGAGTTTGTGCAGGAGATG | AGGCTGAGTGCAAACTTGGT |
| TRAF-6 | GATCGGGTTGTGTGTGTCTG | AGACACCCCAGCAGCTAAGA |
| amastin | GGGGTTCAAAGTTCGAGTGC | AGCAAAGAGCAGCAGCACAG |
| 18SrRNA | TGACTCTAGATAACCTCGGG | GACTCATTCCAATTACAGGG |
| GAPDH | CAGGGGGGAGCCAAAAGGG | CTTGGCCAGGGGTGCTAAGC |
| iNOS | GCAAAAATAGAGGAACATCTGGC | ACCTGATGTTGCCATTGTTGGTG |
| Leishmania parasite specific primer  (Goswami et.al,2020) | CCTATTTTACACCAACCCCCAGT  (JW11) | GGGTAGGGGCGTTCTGCGAAA  (JW12) |
