# Supplementary material for: Leishmania survives by exporting miR-146a from infected to resident cells to subjugate inflammation
Source: Life Sci Alliance. 2022 Feb 24;5(6):e202101229. doi: 10.26508/lsa.202101229 (PMC8881743; doi:10.26508/lsa.202101229)
Supplement: Supplementary file 14 [file LSA-2021-01229_TableS6.docx]

**Table S6 List of Plasmids, siRNAs, miRNA mimics and miRNA inhibitor**

| **Name of Plasmid** | **Source** | **Descripton** |
| --- | --- | --- |
| pmiR122 | As described by Ghosh et. al, 2013 | Plasmid encoding pre-miR-122 under a constitutive U6 promoter |
| HA-HuR | From Dr. Witold Filipowicz | HA-HuR plasmid was cloned in pCIneo backbone and described previously (Kundu et al, 2012) |
| pmiR146a | From Dr. Susanta Roychowdhury | Precursor of miR-146a was cloned in pRNA-U61 vector as described in Sinha et.al, 2011 |
| pRL-Per-miR-122 | Described earlier (Basu and Bhattacharyya, 2014) | Perfect miR-122 binding site downstream of RL coding region |
| Flag-HA-UCP2 | Described earlier (Chakraborty and Bhattacharyya, 2017) | FLAG and HA tagged UCP2 expression plasmid |
| Mito-GFP | From Clonetech | Expression plasmid with GFP protein with Mitochondria targeting sequence |
| YFP-Endo | From Edouard Bertrand | YFP tagged early endosome expression plasmid |
| ER-Ds-Red | From Clonetech | Expression plasmid with Ds-Red protein with ER targeting sequence |
| pRL-Con | From Dr. Witold Filipowicz (Pillai et.al, 2005) | Humanized Renilla Luciferase (RL) coding region |
| pDsRed-Monomer-N1 | From Clonetech | Plasmid encoding DsRed fluorescent protein under CMV promoter |
| control mimic | Ambion® (AM17110) | Pre-miR^TM^miRNA precursor negetive control |
| miR-146a mimic | Ambion®(PM10722) | Pre-miR^TM^miRNA precursor |
| si RNA CON | Dharmacon | ON-TARGETplus SMARTpool siRNA |
| si RNA HuR | Dharmacon | ON-TARGETplus SMARTpool Mouse ELAVL1 |
| Anti-miR-146a-5p | Ambion® (AM10722) | Inhibitor of miR-146a |
| Anti-miR-negative control | Ambion® (AM17010) | Negative control for miRNA inhibitor |
| si RNA RL  (Ghosh et.al, 2015) | From Eurogentec | siRL sense  strand  GCGAGAUCCCUCUCGUUAATT  siRL antisense  strand  UUAACGAGAGGGAUCUCGCGG |
