# Supplementary material for: Leishmania survives by exporting miR-146a from infected to resident cells to subjugate inflammation
Source: Life Sci Alliance. 2022 Feb 24;5(6):e202101229. doi: 10.26508/lsa.202101229 (PMC8881743; doi:10.26508/lsa.202101229)
Supplement: Supplementary file 15 [file LSA-2021-01229_TableS7.docx]

**Table S7 Antibodies used for Western Blot**

| **Antibody name** | **Raised in** | **Dilution used** | **Source** |
| --- | --- | --- | --- |
| Dicer1 | Rabbit | 1:5000 | Bethyl |
| HRS | Rabbit | 1:1000 | Bethyl |
| RAB27a | Rabbit | 1:1000 | Cell Signaling |
| Flotilin 1 | Rabbit | 1:1000 | Cell Signaling |
| Alix | Mouse | 1:200 | Santa Cruz |
| Cytochrome C | Rabbit | 1:1000 | Cell Signaling |
| HuR | Mouse | 1:1000 | Santa Cruz |
| P-p38 | Rabbit | 1:1000 | Cell Signaling |
| P-ERK1/2 | Rabbit | 1:1000 | Cell Signaling |
| HA | Rat Monoclonal | 1:1000 | Roche |
| ERK1/2 | Rabbit | 1:1000 | Cell Signaling |
| P-38 | Rabbit | 1:1000 | Cell Signaling |
| L7a | Rabbit | 1:1000 | Cell Signaling |
| Ago2 | Mouse | 1:1000 | Abnova |
| Calnexin | Rabbit | 1:8000 | Bethyl |
| Rab5 | Rabbit | 1:1000 | Cell Signaling |
| GAPDH | mouse | 1:6000 | Sigma-Aldrich |
| CD63 | Mouse | 1:1000 | BD Pharmingen |
| β-actin | Mouse monoclonal (HRP conjugated) | 1:10000 | Sigma Aldrich |
| UCP2 | Goat | 1:5000 | Novus |
| Rab7 | Rabbit | 1:1000 | Cell Signaling |
| Dynamin 2 | Rabbit | 1:1000 | Cell Signaling |
| RILP | Goat | 1:1000 | Santa Cruz |
| GP63 | Mouse | 1:1000 | LS Biosciences |
